# Mentoring for improving gender equality in academic psychiatry

**DOI:** 10.1192/j.eurpsy.2023.62

**Published:** 2023-07-19

**Authors:** A. Riecher-Rössler

**Affiliations:** Medical Faculty, University of Basel, Basel, Switzerland

## Abstract

**Abstract:**

Although in many countries in the meantime more women than men choose medicine and later psychiatry for their training, key positions in hospitals and research are still mainly held by men. The professional career of women is impeded not only by institutional, but also by psychological barriers such as gender role behavior and gender role stereotypes. Mentoring can help young women to overcome these barriers.

But usually mentoring starts too late. As studies have shown, important decisions about future career steps are taken already towards the end of medical studies. Therefore, gender sensitive teaching and mentoring should start already at university and should not only address young women, but also young men as potential partners and future colleagues - especially regarding their gender role behavior and stereotypes. Mentoring programs considering gender-specific needs should be implemented in the regular teaching during medical studies and in psychiatric training.

Furthermore, women should be coached during their further career steps since there is not only a “glass ceiling” that excludes young women from achieving leadership roles. When they finally have achieved such a role, women often face further difficulties stemming from gender stereotypes and traditional gender roles.

University teachers and employers should be addressed, as well as politicians. Otherwise, psychiatry not only loses a great potential of talents, but might also miss the chance of reforms towards a more gender-sensitive psychiatry and psychotherapy.

**Disclosure of Interest:**

None Declared

